# The Prevalence and Risk Analysis of Cerebral Palsy and Other Neuro-Psychological Comorbidities in Children with Low Birth Weight in Taiwan: A Nationwide Population-Based Cohort Study

**DOI:** 10.3390/jcm13123480

**Published:** 2024-06-14

**Authors:** Hueng-Chuen Fan, Yu-Mei Chang, Jen-Yu Lee, Der-Shiun Wang, Chuan-Mu Chen, Shu-Wei Hu, Kuo-Liang Chiang, Fang-Chuan Kuo

**Affiliations:** 1 Department of Pediatrics, Tungs’ Taichung Metroharbor Hospital, Wuchi, Taichung 435, Taiwan; fanhuengchuen@yahoo.com.tw (H.-C.F.); husw0310@gmail.com (S.-W.H.); 2 Department of Rehabilitation, Jen-Teh Junior College of Medicine, Nursing and Management, Miaoli 356, Taiwan; 3 Department of Life Sciences, Agricultural Biotechnology Center, National Chung Hsing University, Taichung 402, Taiwan; chchen1@dragon.nchu.edu.tw; 4 Department of Pediatrics, School of Medicine, National Defense Medical Center, Taipei 100, Taiwan; okishimashuji317@gmail.com; 5 Department of Pediatrics, Tri-Service General Hospital, Taipei 114, Taiwan; 6 Department of Statistics, Tunghai University, Taichung 407, Taiwan; yumei0115@thu.edu.tw; 7 Department of Statistics, Feng Chia University, Taichung 407, Taiwan; jylee@fcu.edu.tw; 8 Graduate Institute of Clinical Medicine, College of Medicine, National Taiwan University, Taipei 100, Taiwan; 9 The iEGG and Animal Biotechnology Center, Rong Hsing Research Center for Translational Medicine, National Chung Hsing University, Taichung 402, Taiwan; 10 Department of Pediatric Neurology, Kuang-Tien General Hospital, Taichung 433, Taiwan; 11 Department of Nutrition, Hungkuang University, Taichung 433, Taiwan; 12 Department of Physical Therapy, Hungkuang University, Taichung 433, Taiwan

**Keywords:** preterm infants, low birth weight, comorbidities, maternal factors, cerebral palsy, National Health Insurance Research Database

## Abstract

**Background**: This study evaluated early childhood comorbidities of cerebral palsy (CP) in low birth weight (LBW) children and assessed the impact of maternal bio-psychosocial factors on CP risk in preterm infants of varying birth weights (BWs). **Methods**: Data from 15,181 preterm infants (2009–2013) and 151,810 controls were analyzed using Taiwan’s National Health Insurance Research Database. CP prevalence and LBW-associated comorbidities were examined, and odds ratios (ORs) were calculated. **Results**: This study confirmed increasing prematurity and LBW rates in Taiwan, with LBW infants showing higher CP prevalence. Significant maternal risk factors included age extremes (<20 and >40 years). LBW infants exhibited higher risks for respiratory, circulatory, nervous system, and psycho-developmental comorbidities compared with controls, with the lowest BW having even higher ORs. Maternal factors such as family income, the number of hospital admissions, and length of hospital stay were remarkably correlated with BW and subsequent complications. Each additional gestational week crucially reduced the risk of complications in premature infants. **Conclusions**: LBW infants are at a higher risk for CP and various comorbidities, with maternal bio-psychosocial factors playing a critical role. Addressing these factors in prenatal care and interventions is essential to improve outcomes for premature infants.

## 1. Introduction

Cerebral palsy (CP), one of the most prevalent motor disorders affecting children worldwide, results from a static brain lesion in pregnancy or early life [1]. It is characterized by impaired movement and posture, often accompanied by associated conditions such as intellectual impairment, epilepsy, and sensory deficits [2]. It represents a significant public health concern because of its lifelong impact on affected individuals and their families [3,4]. Despite advancements in perinatal care and medical interventions, CP continues to pose substantial challenges to clinical management and has long-term outcomes for affected children [5].

Analysis of the prevalence and risk factors of CP among children with low birth weight (LBW) is crucial for early intervention. While many studies have focused on CP prevalence in preterm populations in Western countries, research on preterm children in Asia is limited [6]. In Taiwan, prematurity rates have risen steadily, increasing from 8.85% to 10.73% between 2004 and 2014 [7]. Wang et al.’s study using Taiwan’s National Health Insurance Research Database (NHIRD) found the highest CP prevalence among extremely low birth weight (ELBW) preterm children (147.3 cases per 1000 neonatal survivors), followed by very low birth weight (VLBW) preterm (97.2 cases) and LBW preterm (27.7 cases) children, with the lowest prevalence among term-born children (2.5 cases) [8].

Low birth weight, defined as a birth weight below 2500 g, is a well-established risk factor for adverse neonatal outcomes, including CP [1]. LBW infants face heightened susceptibility to a myriad of complications stemming from intrauterine growth restriction, prematurity, or a combination [8]. These infants often require specialized care in neonatal intensive care units to mitigate potential morbidities and improve survival rates. Data on preterm populations consistently indicate that the prevalence of CP increases with decreased gestational age or birth weight. However, even for preterm infants at a similar gestational age or birth weight range, the estimations of CP prevalence significantly vary across studies [8,9,10,11].

The relationship between CP and LBW involves complex interactions between biological, environmental, and socioeconomic factors influencing neurodevelopmental outcomes. Epidemiological data consistently identify LBW as a significant CP risk factor, with LBW infants showing a higher CP prevalence than normal birth weight infants. The etiology of CP in LBW infants is multifactorial, involving prenatal, perinatal, and postnatal factors that lead to brain injury and motor impairment [1,10,11,12]. Despite substantial evidence linking LBW and CP, gaps remain in understanding the precise mechanisms and specific risk factors, particularly in non-Western populations, with limited data from Asian countries like Taiwan.

For example, the majority of these studies focused on infants with CP as a single entity, rather than thoroughly analyzing the pathological characteristics and risk factors specific to the LBW population. This study aims to bridge this gap through detailed subgroup analyses, examining the specificity of CP in LBW infants and their related health issues.

Our previous research investigated the prevalence of CP across Taiwan, identifying significant geographical and socioeconomic disparities [13]. Additionally, we analyzed the causes and duration of acute hospitalizations among all severe CP cases from childhood through young adulthood [12]. Therefore, this study focuses on the demographic characteristics of LBW preterm infants and their common comorbidities—particularly CP and other prevalent neuro-psychiatric conditions—and explores maternal factors to identify key influences on these outcomes.

This research utilizes data from the National Health Insurance Research Database (NHIRD) of Taiwan from 2009 to 2013 for a comprehensive analysis of LBW infants, aiming to explore the latest epidemiological trends in CP among this group in Taiwan. This dataset is more current than the one used by Wang et al. from 1998 to 2001 [8], reflecting advancements in medical care and prenatal risk management over the past decade, which are expected to enhance clinical practices and public health strategies. Moreover, this study examines the impacts of maternal age and health on the prevalence of CP and other neurological comorbidities, offering comprehensive risk assessment and management insights.

We hypothesize that prematurity is a significant cause of CP, prompting a detailed investigation into LBW. Through meticulous long-term data analysis, this study thoroughly examines the demographic and sociodemographic factors of LBW infants across various birth weight categories compared to full-term infants. It analyzes their association with five major categories of comorbidities including respiratory, circulatory, neurological, psych-developmental, and nutritional issues, alongside family income. Additionally, this study assesses hospitalization frequency (number of admissions, NOA) and length of stay (LOS) related to birth weight, providing indications of the health conditions of both the mothers and the preterm infants. This comprehensive analysis offers profound insights into the specific health challenges faced by preterm and LBW infants, which are essential for developing effective clinical management strategies and public health policies aimed at supporting these vulnerable groups.

To summarize, this study aims to exclusively analyze the LBW population to deepen our understanding of CP and other relevant diseases in this patient population and to provide rigorous guidance on clinical practice and public health interventions in the future.

## 2. Materials and Methods

In 1995, Taiwan launched the National Health Insurance (NHI) program, designed to provide equal and comprehensive healthcare services to all citizens. Covering approximately 98–99% of Taiwan’s 26 million residents, the NHI system is notable for its excellent accessibility, short waiting times, and relatively low costs [12,13]. The medical data collected through this program are registered in the National Health Insurance Research Database (NHIRD), which serves as an invaluable resource for conducting epidemiological research. This database includes data from nearly the entire Taiwanese population, making it an exceptionally comprehensive source of health information.

The participants were selected from meticulously organized datasets comprising comprehensive patient claims data from January 2009 to December 2013. These datasets encompassed three types of files including ambulatory care claims, inpatient claims, and beneficiary registries, all linked via encrypted but unique personal identification numbers. These files contained crucial information pertaining to patients’ medical diagnoses and demographic attributes. To ensure data accuracy, ambulatory care claims were confined to those sourced from outpatient care datasets specific to medical centers and individuals with severe medical conditions.

The classification of individuals identified as having preterm birth was based on specific codes (765.0x, 765.1x, and V21.3x) from the International Classification of Diseases, Ninth Edition, Clinical Modification (ICD-9-CM), 2001 Edition. These individuals comprised children born prematurely and aged under 6 years in 2009, including infants born prematurely and aged under 1 year in 2010. Patient characteristics, including sex, date of birth, family income, and place of residence, along with admission and visit dates, were meticulously documented.

Preterm children were further categorized into four groups based on birth weight standards outlined in the ICD-9-CM code for premature infants as follows: <999 g; 1000–1499 g; 1500–1999 g; and 2000–2500 g (Table 1). These categories were slightly adjusted from the definitions provided in Wang’s study [8]. Preterm children without any medical records after one month old (i.e., died or moved out of Taiwan) were excluded.

**Figure 1:**
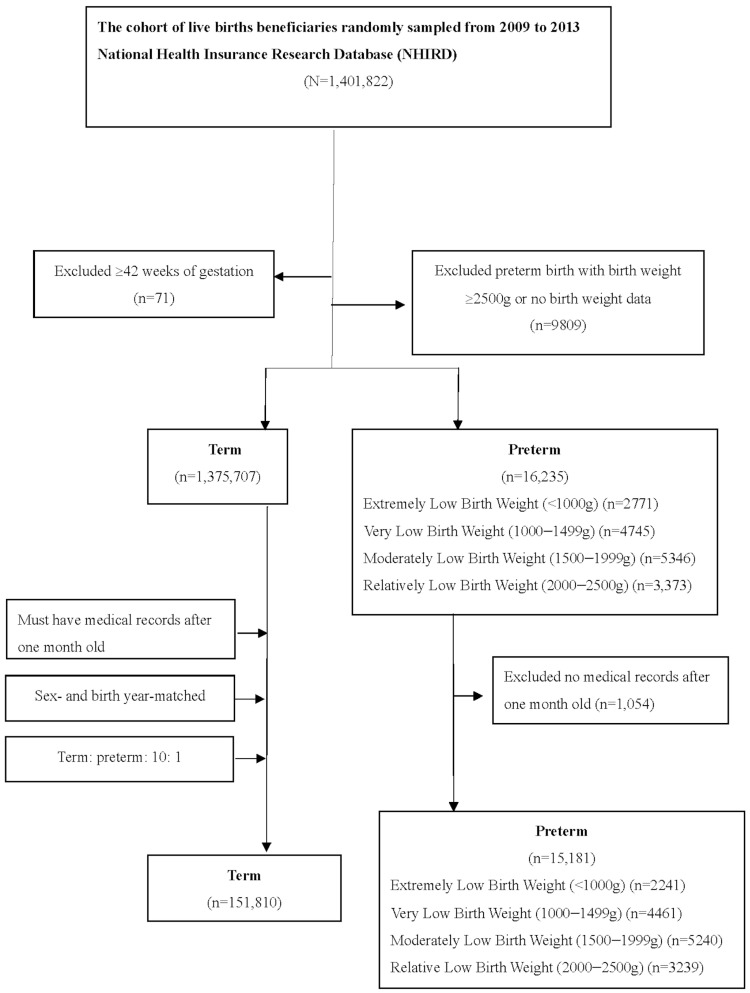
Flowchart of the patient selection process in this study.

The control group consisted of individuals who did not have any preterm birth codes. Similarly, following the same protocol used for preterm children, term-born children with no medical records after one month of age were also excluded. Control groups were matched with prematurity groups throughout the study period. Each premature infant was carefully matched by age and sex with 10 controls. Participants lacking medical records, both preterm and term-born children, were excluded from this study (Figure 1).

### 2.1. Definition of Comorbidities

Comorbidities of each individual were verified to ensure a comprehensive examination of childhood health conditions. All enrolled individuals were included in the analysis to provide a thorough investigation. Making use of the unique identification number of each participant, we analyzed their inpatient and outpatient service records within the NHIRD for the presence of common comorbidities, as each medical claim record can contain additional ICD-9-CM codes. A comprehensive literature review informed the compilation of a list of comorbidities associated with preterm birth; their diagnostic codes are detailed in Supplementary Table S2. In addition, data on the number of admissions (NOA) and length of hospital stay (LOS) were collected for each participant and control.

To ensure the accuracy of cerebral palsy (CP) diagnosis and enable comparison with prior studies [8,13,14], we defined CP as children diagnosed with infantile cerebral palsy (ICD-9-CM 343). Furthermore, these children needed to have undergone rehabilitation, been registered in the catastrophic registry, or been diagnosed with infantile CP by pediatricians or rehabilitation physicians based on at least three outpatient department visit claims within a year. Children meeting these criteria but subsequently diagnosed with progressive neurological disorders (ICD-9-CM codes 330, 331.0–331.2, 331.7–331.9, 334, 335, 340, 341) or spina bifida (741) were excluded from the CP group.

### 2.2. Maternal Biopsychosocial Factors

Information on family income, maternal age at childbirth (MACB), and maternal NOA and LOS was obtained using the personal identification numbers of enrolled individuals and controls, which are linked to a beneficiary registry enabling indirect access to maternal data. Four maternal groups were characterized based on MACB as follows: <20 years, 20–29 years, 30–39 years, and ≥40 years. This stratification facilitated a comprehensive analysis of maternal influences on child health outcomes.

### 2.3. Statistical Analysis

All data processing procedures, including data selection, merging, and aggregation, as well as statistical analyses, were conducted using the R Project for Statistical Computing (version 3.6.2). The chi-square test was applied to examine both categorical and continuous demographic variables, including birth weight, sex, family income, and place of residence, of the enrolled individuals and controls. Additionally, the binomial test was used to compare the proportions of CP prevalence in each premature group with the control group. A significance level of *p* ≤ 0.05 was used.

To evaluate comorbidities, the chi-square test was initially used to assess differences in the proportions of comorbidities among the various birth weight groups. Subsequently, conditional logistic regression was employed to compute sex- and age-stratified odds ratios (ORs) for each comorbidity of the birth weight groups relative to the control group, providing nuanced insights into these associations. Furthermore, logistic regression was employed to determine the ORs of each comorbidity for various income levels compared to the control group. In addition, logistic regression facilitated the calculation of MACB-stratified ORs for various birth weight groups relative to the control group and offered a comprehensive understanding of the impact of maternal age, comorbidities, and birth complications.

Maternal and infant outcomes, including the NOA and LOS, were assessed using analysis of variance (ANOVA) to examine the differences among birth weight groups, with the addition of Pearson correlation coefficients to measure the strength of association. Where ANOVA assumptions were not met, alternative statistical methods such as the Kruskal–Wallis test and Welch’s ANOVA were employed to evaluate medians rigorously. This analysis aimed to determine if LBW preterm infants were more frequently hospitalized or had longer hospital stays, revealing the direct impact of weight on their hospitalization needs. Additionally, it explored whether frequent hospitalization and longer stays in LBW infants were related to maternal factors such as genetic predisposition, chronic health conditions, or the stress of caregiving. Poor maternal health conditions may have led to weaker preterm infants, providing insights for future preterm birth prevention.

## 3. Results

### 3.1. Descriptive Statistics of Demographic Characteristics

Table 2 presents the demographic characteristics of the enrollees and controls. A total of 15,181 enrollees and 151,810 controls were included in the analysis. The birth weight distribution of enrollees was as follows: 14.76% had a birth weight of <1000 g; 29.39% were in the range of 1000–1499 g; 34.52% were 1500–1999 g; and 21.34% were 2000–2500 g. Both were intentionally matched for age and sex, resulting in 46.89% females and 53.11% males in each group. The analysis showed discrepancies in household income between the groups, with a higher proportion of the enrollees in the lowest income level (22.71%) compared with the controls (11.54%). Conversely, a smaller proportion of the enrollees were in the highest income level (12.86%) compared with the controls (15.71%). This difference was statistically significant (*p* < 0.0011). The geographic distribution also differed between the two groups. While 47.84% of the enrollees were born in northern Taiwan, this proportion was slightly higher for the controls (53.93%). Conversely, the enrollees were less represented in the central, southern, eastern, and off-island regions compared with the controls. The differences in the geographic distribution were statistically significant (*p* < 0.0011), indicating regional disparities between the enrollees and controls.

The distribution of maternal age showed significant differences between the two groups (*p* < 0.001). The percentages of mothers aged less than 20 years were 0.77% and 0.57%, aged 20–30 years were 27.66% and 34.99%, aged 30–40 years were 67.33% and 61.96%, and aged 40 years and above were 4.24% and 2.48% for the enrollees and controls, respectively.

### 3.2. Comorbidities of Premature Infants Stratified by Family Income Levels

Table 3 presents the ORs for various comorbidities stratified by family income levels, using logistic regression, with the highest income bracket (new Taiwan dollars [NTD] >50,000) as the reference group. In the lowest income bracket (NTD 0–10,000), participants showed significantly higher odds of respiratory (OR = 2.31; 95% CI: 2.11–2.51), circulatory (OR = 2.68; 95% CI: 2.27–3.10), psycho-developmental (OR = 2.61; 95% CI: 2.25–2.97), and gastrointestinal and nutritional (OR = 1.51; 95% CI: 1.29–1.73) comorbidities compared with the highest income group. In the NTD 10,001–25,000 income bracket, participants also exhibited increased odds of respiratory (OR = 1.21; 95% CI: 1.12–1.31), circulatory (OR = 1.53; 95% CI: 1.34–1.73), and psycho-developmental (OR = 1.50; 95% CI: 1.32–1.67) comorbidities. These findings underscore a significant link between family income levels and the prevalence of comorbidities in infants born prematurely, highlighting that lower incomes correlate with higher ORs for these health issues. Notably, the relationship was particularly strong for respiratory, circulatory, and psycho-developmental conditions in the lower income brackets. Conversely, the association between income level and neurological comorbidities was relatively weak.

### 3.3. Common Neuro-Psychological Comorbidities in Infants Born Prematurely and the Association with Birth Weight

Table 4 presents the ORs of neurological comorbidities for different LBW groups compared to the normal control group in Taiwan between 2009 and 2014. In the case of congenital brain anomalies, all LBW groups had significantly higher odds compared with the normal control group. The highest OR was observed for the <1000 g group (OR: 6.55; 95% CI: 5.30–8.09), followed by the 1000–1499 g group (OR: 6.14; 95% CI: 5.24–7.20), 1500–1999 g group (OR: 5.54; 95% CI: 4.75–6.47), and 2000–2499 g group (OR: 6.35; 95% CI: 5.31–7.59). Similarly, for CP, the OR decreased with increasing birth weight. The highest OR was observed for the <1000 g group (OR: 11.80; 95% CI: 9.42–14.79), followed by the 1000–1499 g group (OR: 5.53; 95% CI: 4.41–6.93), 1500–1999 g group (OR: 3.36; 95% CI: 2.57–4.38), and 2000–2499 g group (OR: 1.47; 95% CI: 0.90–2.39).

Similar trends were observed for other neurological comorbidities such as hydrocephalus, stroke, ophthalmic problems, and epilepsy, with significantly higher ORs for lower birth weight groups compared with the control. Certain comorbidities such as movement disorders, sleep disorders, and anxiety showed decreasing ORs with increasing birth weight. These findings underscore the association between LBW and increased risk of neurological comorbidities in children.

**Figure 2:**
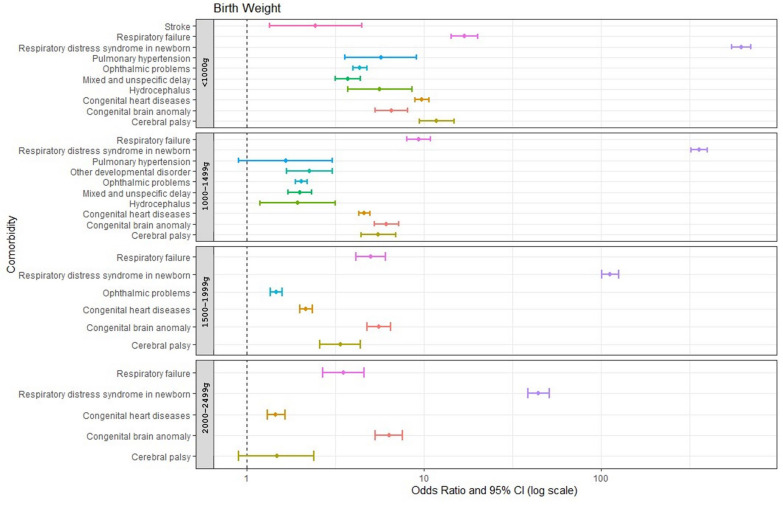
Comorbidities with odds ratio > 1 in different birth weight groups compared with the control group.

Figure 2 illustrates all comorbidities with an OR greater than 1 for various birth weight groups relative to the control group. It demonstrates a correlation wherein lower birth weight of prematurely born infants is correlated with a higher number of comorbidities with an OR greater than 1 compared with the control group. All infants born prematurely were associated with higher odds ratios of respiratory distress syndrome (RDS), congenital brain anomalies, respiratory failure, cerebral palsy, and congenital heart diseases. Notably, RDS and respiratory failure showed a trend in which lower birth weight was correlated with a significantly higher OR. In addition, all infants born prematurely with a birth weight below 2000 g experienced ophthalmic problems. Those with a birth weight below 1500 g showed significantly more comorbidities, including mixed developmental delay, hydrocephalus, and pulmonary hypertension, compared with those with a birth weight of 1500 g or higher. Specifically, the <1000 g group showed a higher OR for stroke (2.44) compared with the 1000–1499 g group.

**Figure 3:**
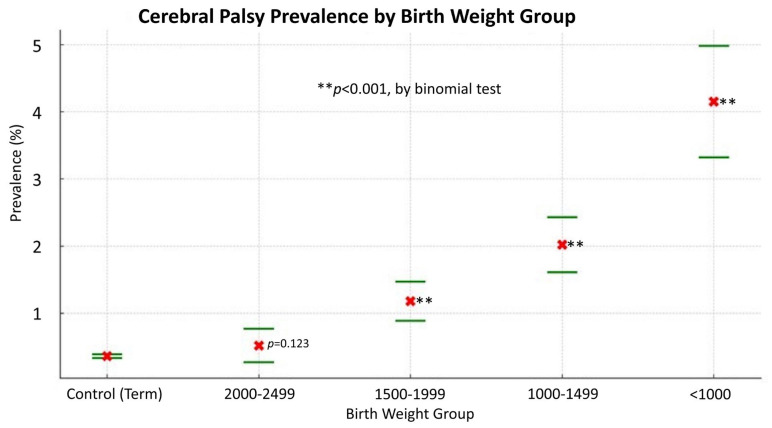
The prevalence of cerebral palsy (CP) under different birth weights. These results demonstrate a significant inverse relationship between the prevalence of CP and birth weight, particularly highlighting the high prevalence rate of CP among infants with low birth weight.

Figure 3 reveals a notable inverse correlation between the prevalence of CP and birth weight, highlighting the substantially higher prevalence of CP in babies with lower birth weights. Infants born under 1000 g have an over 11-fold increased risk of CP compared with those born weighing 2500 g or more. Each ascending birth weight category (1000–1499 g, 1500–1999 g, and 2000–2499 g) shows a decreased risk, demonstrating a clear link between higher birth weight and reduced CP prevalence. The binomial test results indicate a high level of statistical significance (*p* < 0.001) for the lower weight categories compared with the control group, confirming the robustness of this association. However, the 2000–2499 g group does not show a statistically significant difference from the control group (*p* = 0.123).

### 3.4. Inpatient Care Use

Table 5 presents the admission frequency and LOS in hospital of both preterm infants and mothers, categorized by birth weight group. For preterm infants, the mean NOA increased with decreasing birth weight. Specifically, infants weighing less than 1000 g had the highest mean NOA (2.80, SD = 2.67), followed by those in the 1000–1499 g (2.35, SD = 2.30), 1500–1999 g (2.23, SD = 2.27), and 2000–2499 g (2.30, SD = 2.32) groups. The NOA of these groups was significantly higher than that of the control group (mean NOA = 2.00, SD = 1.89). Statistical analysis indicated a significant difference in the NOA between the birth-weight groups (*p* < 0.001).

Similarly, there was a decreasing trend in LOS in hospital of preterm infants with increasing birth weight. Infants weighing less than 1000 g had the longest mean LOS (16.85 days, SD = 26.07), followed by the 2000–2499 g (13.05 days, SD = 23.25), 1000–1499 g (12.11 days, SD = 17.55), and 1500–1999 g (12.01 days, SD = 21.28) groups. The LOS of these groups was significantly higher than that of the control group (mean LOS = 8.80 days, SD = 12.96). The differences in LOS among the birth weight groups were statistically significant (*p* < 0.001).

Furthermore, similar trends were observed for mothers in both the NOA and LOS. Mothers of infants with lower birth weights had a higher mean NOA and longer LOS compared with mothers of infants with higher birth weights and the control group. Statistical analysis revealed significant differences in both the NOA and LOS between the birth-weight groups (*p* < 0.001). These findings underscore the increased healthcare use and duration of hospitalization of both preterm infants and their mothers, particularly infants with lower birth weights and their mothers, necessitating comprehensive care and support strategies for this vulnerable population.

### 3.5. NOA and LOS Correlations for Preterm Infants and Mothers

**Figure 4:**
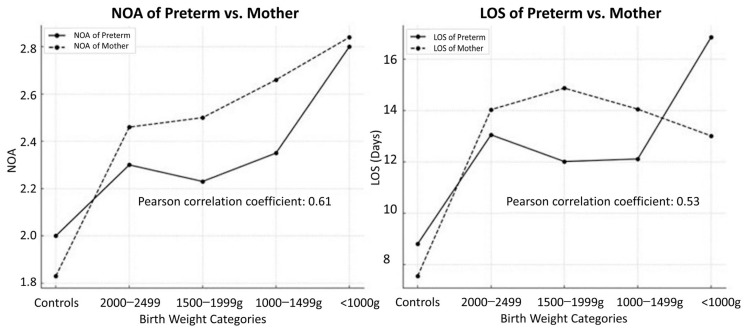
Correlations between birth weight and the number of admissions (NOA) of preterm infants and mothers and between birth weight and length of stay (LOS) in hospital of preterm infants and mothers, suggesting that there is a significant correlation between birth weight and both the NOA and LOS in hospital for preterm infants and mothers.

Figure 4 illustrates correlations between NOA and birth weight categories and the positive correlation (Pearson r = 0.61, *p* < 0.001) between the NOA of preterm infants and mothers and different birth weight categories. As birth weight decreased, the NOA of pre-term infants and that of their mothers showed an upward trend, indicating a potential association between maternal and infant healthcare use. Furthermore, the relationship between the LOS of preterm infants (Pearson r = 0.53, *p* < 0.001) and the NOA of mothers across birth weight categories was explored. A similar positive correlation was evident, with lower birth weight categories associated with a longer LOS of preterm infants and a higher NOA of mothers. This trend suggests that infants born with lower birth weights may require more extended hospital stays, potentially leading to increased healthcare use by their mothers.

Overall, these findings underscore the interconnectedness of maternal and infant healthcare experiences, highlighting the importance of considering both maternal and infant factors in the management of preterm birth and associated healthcare needs.

### 3.6. Association between Low Birth Weight and Maternal Age

Table 6 presents the association between LBW and MACB, revealing noteworthy trends across different maternal age groups. Among the enrollees, LBW infants exhibited different ORs across maternal age categories. The ORs of infants weighing less than 1000 g of mothers aged <20 years (OR: 2.29; 95% CI: 1.07–4.92) and mothers aged ≥40 years (OR: 2.57; 95% CI: 1.78–3.72) were significantly elevated. Similarly, the ORs of infants weighing 1000–1499 g of both the younger and older maternal age groups were elevated. Notably, the OR of infants weighing 2000–2499 g of mothers aged ≥40 years showed an increase (OR: 2.68; 95% CI: 2.04–3.51). Conversely, no significant associations were observed between LBW and maternal age in the case of the control group. These findings underscore the complex interplay between LBW and maternal age, suggesting differential risk patterns across LBW categories and maternal age groups.

## 4. Discussion

In this nationwide population-based cohort study conducted in Taiwan, we aimed to investigate the prevalence of CP and its associated risk factors among children with LBW. Our findings focus on the epidemiology of CP in this vulnerable population and provide valuable insights for clinical practice and public health interventions.

Our findings revealed a notably higher prevalence of CP among children with LBW compared with the general population. This finding is consistent with previous research, which has consistently identified LBW as a significant risk factor for CP development [15,16,17]. A cohort study by Wang et al. using Taiwan’s NHIRD data reported LBW and extreme prematurity as notable risk factors for CP in Taiwanese children [8]. Kim et al. also found that LBW prevalence rose from 1.40% in 2007 to 2.09% in 2011, representing a nearly 1.5-fold increase. In addition, the proportion of individuals with a history of prematurity or LBW among those with CP increased from 29.8% in 2007 to 37.7% in 2011 [18]. Moreover, the findings of our study highlighted an inverse correlation between CP incidence and birth-weight categories, further supporting the established association between LBW and CP. This result underscores the pivotal role of birth weight as a predictive factor for CP in children and emphasizes the urgent need for targeted screening and intervention strategies for LBW infants.

We found significant regional differences in the distribution of participants compared with the control group. These disparities are likely due to variations in the availability of medical facilities, socioeconomic status, and population density across different areas of Taiwan. For instance, in the northern region, particularly in metropolitan areas such as Taipei, New Taipei, and Taoyuan, the inclusion of more low birth weight infants and control groups is facilitated by the high population density and abundance of medical resources. Additionally, these areas may exhibit a relatively lower proportion of LBW infants because of more frequent public health activities and the presence of specialized medical institutions. Conversely, in the central and southern regions, where socioeconomic status is generally lower than in the north, residents may face more barriers in accessing health resources, which could explain the higher occurrence of LBW infants in these areas, reflecting regional imbalances. In the eastern region, the smaller resident population, lower fertility rates, and the presence of many Taiwanese indigenous people with low socioeconomic status, coupled with a severe scarcity of medical resources, highlight significant disparities in medical access and population distribution between urban and rural areas, resulting in a higher proportion of preterm births. However, the outlying islands are underrepresented because of their low population. These regional disparities have also been noted in previous studies on the prevalence of epilepsy and CP in Taiwan [13,19]. Study results emphasize the socioeconomic disparities in the prevalence of specific comorbidities in prematurely born infants and underscore the importance of addressing socioeconomic factors in healthcare interventions aimed at reducing the burden of these conditions in vulnerable populations.

Our study also found that prematurely born infants faced an increased risk of various comorbidities, which affected their respiratory, circulatory, and nervous systems and psychological development. Similar findings have been reported by other studies, indicating that preterm birth is linked to increased risks of diverse multimorbidity patterns [12,20,21]. In addition, Chang et al.’s study of a Taiwanese cohort revealed that preterm babies were more susceptible to mortality and the development of morbidities, with patients with CP being particularly vulnerable to various diseases [22]. This trend underscores the significant impact of birth weight on the likelihood of developing CP, with lower birth weights being correlated with a higher prevalence of this neurological condition. This finding emphasizes the critical role of birth weight as a predictive factor for CP development and underscores the importance of early interventions and tailored care for infants with lower birth weights to mitigate the risk of CP.

Furthermore, our study investigated the impact of socioeconomic factors on CP prevalence among LBW infants. We observed a significant association between family income level and the likelihood of psycho-developmental comorbidities among LBW infants. Previous studies have consistently highlighted the vulnerability of premature infants to lifelong neurological conditions such as CP, autism spectrum disorder, learning disability, and cognitive or developmental delays [23,24]. Our findings are similar to those of a systematic review conducted by Solaski et al., which identified low socioeconomic status as a significant risk factor for increased CP prevalence [25]. Similarly, a study conducted in Korea revealed a marked discrepancy in CP incidence between affluent and deprived groups, with the latter experiencing significantly higher rates. Specifically, lower family income was correlated with a higher risk of psycho-developmental comorbidities, emphasizing the role of socioeconomic status in shaping health outcomes in vulnerable populations [18].

We found some interesting results after analyzing the neuro-psychological comorbidities in infants with LBW. In particular, infants with a body weight below 1000 g had a significantly lower incidence of motor impairment than the control group of healthy infants (OR = 0.17), while infants who weighed between 2000 and 2499 g had an approximately zero incidence of sleep disorders (OR = 0.10). However, these results may be influenced by restrictions in clinical documentation. The national health insurance system in Taiwan limits the maximum diagnosis codes to three and the hospital stay records to five for every piece of outpatient data. This restriction particularly affects patients with chronic and severe health conditions such as preterm and CP infants because the diagnosis codes involved are often recorded first because of their clinical importance. Consequently, less pressing health problems such as sleep disorders or motor impairments may not be documented because of documentation priority. This prioritization may restrict the documentation of other secondary but clinically important comorbidities. Therefore, a low odds ratio may reflect documentation bias rather than actual disease prevalence. Further studies should take into account this restriction and verify our findings by exploring other possible data sources or enriching the data. This analytical restriction also highlights the importance of improving health information systems, particularly in diagnosis code documentation, as it is pivotal for accurately showcasing a patient’s whole-person health status.

Our analysis of healthcare patterns also sheds light on the needs of LBW infants and their mothers. We found a correlation between birth weight categories and preterm infant admissions and maternal admissions, suggesting intertwined healthcare use. LBW categories were associated with longer preterm infant hospital stays and more maternal admissions, emphasizing the need for comprehensive support strategies for LBW infants and their mothers. These findings align with those of previous studies showing that longer hospital stays are linked to LBW [26,27]. Mehretie et al.’s cross-sectional study further linked gestational age, complications, and initial management with the duration of hospital stay of VLBW preterm neonates [28]. This highlights the importance of coordinated care and multidisciplinary interventions for addressing the complex healthcare needs of LBW infants and their families.

Our analysis also explored the relationship between the prevalence of LBW and maternal age. Interestingly, we found that infants with LBW born to mothers under 20 years or over 40 years exhibited significantly higher odds of LBW compared with those born to mothers aged 20–30 years. A Taiwanese cohort study presented similar results, revealing that the maternal age of 25–29 years was associated with the lowest risk of preterm birth, followed by 30–34 years. In contrast, the >35-year age group showed increased odds [7,29]. These findings suggest that extremes in maternal age may contribute to the heightened risk of LBW and adverse outcomes, hinting at maternal age as a risk factor for adverse pregnancy outcomes and neurodevelopmental disorders, such as CP, in offspring [30,31,32].

The strengths of our study are its large sample size, population-based design, and comprehensive analysis of risk factors and healthcare-use patterns, providing robust evidence for our findings. The use of nationwide data allowed us to capture a representative sample of LBW infants and their mothers. Moreover, our study employed standardized diagnostic criteria for CP and rigorous statistical methods to adjust for potential confounders, which enhanced the validity and reliability of our results.

Despite these strengths, our study has some limitations that warrant consideration. The retrospective nature of the study design limited our ability to establish causal relationships between risk factors and LBW outcomes. In addition, the reliance on an administrative database might have introduced biases related to data completeness and accuracy. Furthermore, the lack of detailed clinical information, such as gestational age and severity of CP, limited our ability to perform subgroup analyses and explore potential effect modifiers.

A limitation of this study is the potential impact of constraints within the original health insurance database diagnostic code records. Specifically, the upper limit for diagnostic codes recorded per outpatient visit is three, and for hospitalization records, it is five. This limitation could result in an incomplete depiction of participants’ health conditions within the dataset. Moreover, diagnoses such as preterm birth and cerebral palsy must be consistently recorded because of reimbursement implications, thereby restricting physicians from documenting other non-primary yet clinically significant conditions. Particularly in developmental assessment clinics, where cases of preterm birth with cerebral palsy might already be diagnosed with developmental delay, the recording of additional symptoms like sleep problems or motor disorders may be overlooked in health insurance data, potentially skewing the analysis.

This study significantly contributes to the understanding of CP prevalence among and risk factors of children with LBW in Taiwan. It underscores the importance of early identification and intervention strategies targeting LBW infants, particularly those born to mothers in extreme age groups or from disadvantaged socioeconomic backgrounds.

Recommendations and future research

Based on our study, we recommend enhancing the care for children with LBW and CP in Taiwan through early screening and intervention. This includes implementing standardized protocols to detect CP in LBW infants and providing prompt access to physical, occupational, and speech therapy. Measures strengthening maternal and infant healthcare services, addressing socioeconomic disparities, promoting healthcare integration, and raising public awareness about CP are also crucial. These measures aim to optimize outcomes and support families facing the challenges of caring for children with LBW and CP.

Future research should focus on elucidating the underlying mechanisms linking LBW to CP and developing tailored interventions to mitigate the CP risk of vulnerable populations. In addition, prospective studies with longitudinal follow-up are needed to further elucidate the long-term outcomes and trajectories of LBW infants with CP. By addressing these gaps in knowledge, we can enhance early intervention efforts and improve outcomes for children with LBW and CP.

## 5. Conclusions

In conclusion, our nationwide population-based cohort study provides valuable insights into the prevalence of and risk factors associated with CP among children with LBW in Taiwan. Our findings highlight the increased risk of CP among LBW infants and underscore the importance of early detection, intervention, and comprehensive healthcare support for this vulnerable population. By identifying maternal age, family income level, and healthcare use patterns as significant predictors of CP among LBW infants, our study informs targeted preventive strategies to mitigate the risk factors and reduce the incidence of CP. Concerted efforts from healthcare providers, policymakers, and community stakeholders are needed to implement evidence-based preventive measures and support programs to address the complex needs of children with LBW, with the ultimate goal of preventing CP in this high-risk group. Collaborative efforts and proactive initiatives can enhance preventive care and promote better developmental outcomes for children with LBW, thereby reducing their likelihood of developing CP and improving their long-term prognosis.

## Figures and Tables

**Table 1:** ICD 9 codes and subcategories of premature children enrolled in this study.

Subcategories of Preterm Birth or Low Birth Weight	ICD 9 Codes
Extremely preterm (<1000 g)	765.01, 765.02, 765.03, 765.11, 765.12, 765.13, V21.31, V21.32
Very preterm (1000–1499 g)	765.04, 765.05, 765.14, 765.15, V21.33
Moderately preterm (1500–1999 g)	765.06, 765.07, 765.16, 765.17, V21.34
Late preterm (2000–2499 g)	765.08, 765.18, V21.35

ICD: International Classification of Diseases.

**Table 2:** Demographic characteristics of the enrollees and controls.

	Enrollees *n* (%)	Controls *n* (%)	*p*-Value
Birth weight			
Total sample size	15,181	151,810	
<1000 g	2241 (14.76)		
1000–1499 g	4461 (29.39)		
1500–1999 g	5240 (34.52)		
2000–2500 g	3239 (21.34)		
Sex			
Female	7119 (46.89)	71,190 (46.89)	
Male	8062 (53.11)	80,620 (53.11)	1.000 ^1^
Income			
1 (Lowest)	3447 (22.71)	17,525 (11.54)	
2	5756 (37.92)	61,905 (40.78)	
3	4026 (26.52)	48,524 (31.96)	
4 (Highest)	1952 (12.86)	23,856 (15.71)	<0.001 ^2^
Location			
Northern	7263 (47.84)	81,875 (53.93)	
Central	3661 (24.12)	32,401 (21.34)	
Southern	3982 (26.23)	35,252 (23.22)	
Eastern	267 (1.76)	2032 (1.33)	
Off-island	8 (0.05)	250 (0.18)	<0.001 ^2^
Maternal age			
<20 years	42 (0.77)	331 (0.57)	
20–30 years	1512 (27.66)	20,286 (34.99)	
30–40 years	3680 (67.33)	35,921 (61.96)	
≥40 years	232 (4.24)	1436 (2.48)	<0.001 ^2^

^1^ matched by sex and age, ^2^ chi-square test for independence

**Table 3:** Comorbidities of premature infants stratified by family income levels.

Comorbidities	Income Level (in NTD)	Enrollees	Controls	OR	95% CI
	0–10,000 (lowest)	1506	9860	2.31	(2.11, 1.31)
	10,001–25,000	3039	37,857	1.21	(1.12, 1.31)
Respiratory	25,001–50,000	2003	28,374	1.07	(0.98, 1.16)
	>50,001 (highest)	872	13,197	---	---
	0–10,000 (lowest)	510	1031	2.68	(2.27, 3.10)
	10,001–25,000	1343	4756	1.53	(1.34, 1.73)
Circulatory	25,001–50,000	906	3812	1.29	(1.12, 1.46)
	>50,001 (highest)	360	1954	---	---
	0–10,000 (lowest)	72	693	1.10	(0.75, 1.45)
	10,001–25,000	321	3250	1.05	(0.80, 1.29)
Neurological	25,001–50,000	205	2314	0.94	(0.71, 1.17)
	>50,001 (highest)	105	1112	---	---
	0–10,000 (lowest)	567	1835	2.61	(2.25, 2.97)
	10,001–25,000	1459	8218	1.50	(1.32, 1.67)
Psycho-developmental	25,001–50,000	964	6513	1.25	(1.10, 1.40)
	>50,001 (highest)	417	3517	---	---
	0–10,000 (lowest)	405	6539	1.51	(1.29, 1.73)
	10,001–25,000	1091	26,420	1.01	(0.88, 1.13)
GI and nutritional	25,001–50,000	721	19,508	0.90	(0.78, 1.02)
	>50,001 (highest)	362	8821	---	---

NTD: new Taiwan dollars, ‘---’ indicates the reference group.

**Table 4:** Neuro-psychological comorbidities in each low birth weight group compared to the normal control group in Taiwan (2009–2014).

Comorbidities	Birth Weight Group			
	<1000 g (OR, 95% CI)	1000–1499 g (OR, 95% CI)	1500–1999 g (OR, 95% CI)	2000–2499 g (OR, 95% CI)
Neurological:	4.27 (3.92, 4.65)	2.08 (1.94, 2.23)	1.53 (1.42, 1.64)	1.11 (1.01, 1.22)
Congenital brain anomaly	6.55 (5.30–8.09)	6.14 (5.24–7.20)	5.54 (4.75–6.47)	6.35 (5.31–7.59)
Cerebral palsy	11.80 (9.42–14.79)	5.53 (4.41–6.93)	3.36 (2.57–4.38)	1.47 (0.90–2.39)
Hydrocephalus	5.63 (3.71–8.54)	1.93 (1.18–3.16)	0.87 (0.44–1.70)	0.61 (0.22–1.68)
Stroke	2.44 (1.34–4.44)	0.87 (0.43–1.74)	0.46 (0.19–1.11)	0.44 (0.14–1.33)
Ophthalmic problems	4.34 (3.96–4.75)	2.02 (1.87–2.19)	1.46 (1.35–1.58)	0.94 (0.84–1.06)
Hearing problems	1.12 (0.83–1.51)	0.82 (0.64–1.05)	0.42 (0.31–0.58)	0.17 (0.10–0.31)
Epilepsy	0.99 (0.74–1.39)	0.53 (0.39–0.72)	0.43 (0.32–0.59)	0.38 (0.25–0.57)
Movement disorders	0.17 (0.06–0.54)	0.20 (0.10–0.41)	0.10 (0.04–0.25)	0.04 (0.01–0.17)
Sleep disorders	0.44 (0.11–1.67)	0.22 (0.06–0.82)	0.10 (0.02–0.57)	0.00 (0.00–0.60)
Psycho-developmental:	2.09 (1.82–2.41)	1.27 (1.12–1.44)	0.62 (0.53–0.73)	0.35 (0.27–0.45)
Developmental delay	2.69 (2.32–3.12)	1.58 (1.39–1.80)	0.78 (0.67–0.92)	0.43 (0.33–0.56)
Mixed and nonspecific delay	3.70 (3.14–4.36)	1.99 (1.71–2.31)	0.94 (0.77–1.14)	0.44 (0.31–0.62)
Other developmental disorder	2.44 (1.64–3.63)	2.25 (1.67–3.02)	1.14 (0.79–1.66)	0.83 (0.49–1.41)
Speech or language developmental disorder	0.73 (0.51–1.04)	0.45 (0.33–0.60)	0.30 (0.21–0.43)	0.21 (0.12–0.35)
Developmental coordination disorder	0.69 (0.27–1.74)	0.75 (0.40–1.42)	0.36 (0.15–0.85)	0.00 (0.00–0.00)
Learning disability	1.49 (0.37–6.00)	1.11 (0.36–3.45)	0.00 (0.00–0.00)	0.00 (0.00–0.00)
Childhood emotional disturbances	0.00 (0.00–0.00)	0.12 (0.02–0.68)	0.11 (0.02–0.57)	0.00 (0.00–0.00)
ADHD	0.33 (0.13–0.79)	0.25 (0.13–0.50)	0.21 (0.10–0.44)	0.15 (0.05–0.44)
Intellectual disabilities	0.34 (0.05–2.26)	0.51 (0.17–1.54)	0.00 (0.00–0.00)	0.00 (0.00–0.00)
Autistic spectrum disorder	0.37 (0.10–1.34)	0.26 (0.09–0.78)	0.23 (0.08–0.70)	0.00 (0.00–0.00)
Anxiety	0.00 (0.00–0.00)	0.00 (0.00–0.00)	0.22 (0.03–1.58)	0.00 (0.00–0.00)
Acute stress reaction	1.80 (0.25–13.24)	0.00 (0.00–0.00)	0.00 (0.00–0.00)	0.00 (0.00–0.00)

**Table 5:** Admission frequency (NOA) and length of stay (LOS) in hospital of preterm infants and their mothers.

	Birth Weight	Mean	Standard Deviation	Number of Patients	*p*-Value ^3^
NOA ^1^ of preterm infants	<1000 g	2.80	2.67	1243	<0.001
	1000–1499 g	2.35	2.30	2045	
	1500–1999 g	2.23	2.27	2153	
	2000–2499 g	2.30	2.32	1306	
	Control	2.00	1.89	79,466	
LOS ^2^ of preterm infants	<1000 g	16.85	26.07	1243	<0.001
	1000–1499 g	12.11	17.55	2045	
	1500–1999 g	12.01	21.28	2153	
	2000–2499 g	13.05	23.25	1306	
	Control	8.80	12.96	79,466	
NOA of mothers	<1000 g	2.84	1.79	758	<0.001
	1000–1499 g	2.66	1.97	1561	
	1500–1999 g	2.50	1.53	1911	
	2000–2499 g	2.46	1.51	1236	
	Control	1.83	1.15	57,974	
LOS of mothers	<1000 g	13.01	13.55	758	<0.001
	1000–1499 g	14.05	16.08	1561	
	1500–1999 g	14.87	16.64	1911	
	2000–2499 g	14.03	14.01	1236	
	Control	7.55	8.20	57,974	

^1^ Number of admissions; ^2^ length of stay; ^3^ *p*-values for the comparisons of group means using ANOVA, Kruskal–Wallis test, and Welch’s ANOVA are all less than 0.001.

**Table 6:** Odds ratios (ORs) for associations between the low birth weight of infants and maternal age.

	Maternal Age							
	<20 Years		20–30 Years		30–40 Years		≥40 Years	
Birth weight	n, OR ^1^ (95% CI)							
Enrollees	42	1.70 (1.23–2.36)	1512	---	3680	1.37 (1.29–1.46)	232	2.17 (1.86–2.51)
<1000 g	7	2.29 (1.07–4.92)	187	---	530	1.60 (1.35–1.89)	34	2.57 (1.78–3.72)
1000–1499 g	16	2.25 (1.35–3.75)	436	---	1044	1.35 (1.21–1.51)	65	2.11 (1.61–2.75)
1500–1999 g	11	1.23 (0.67–2.26)	546	---	1286	1.33 (1.20–1.47)	68	1.76 (1.36–2.28)
2000–2499 g	8	1.43 (0.70–2.91)	343	---	820	1.35 (1.19–1.53)	65	2.68 (2.04–3.51)
Control group	331	---	20,286	---	35,921	---	1436	---

^1^ The reference group for OR calculation was the 20–30-year age group for maternal age and the control group for birth weight groups.

## Data Availability

The following information is provided regarding data availability: The data are available from the National Health Insurance Research Database (NHIRD) published by the Taiwan National Health Insurance (NHI) Bureau. The data utilized in this study cannot be made available in this paper, the supplemental files, or in a public repository because of the “Personal Information Protection Act” executed by Taiwan’s government, starting in 2012. Requests for data can be sent as a formal proposal to the NHIRD (http://nhird.nhri.org.tw (accessed on 20 April 2024)) or by email to nhird@nhri.org.tw.

## Supplementary Materials

The following supporting information can be downloaded at: https://www.mdpi.com/article/10.3390/jcm13123480/s1, Table S1: title: Sub-categories of enrollees of prematurity and their ICD 9 codes. Table S2: title: Disease diagnostic coding of comorbidities. Table S3: title: The number and proportion of subsequent comorbidities in different birth weight groups of premature infants and the results of the chi-square test. Table S4: title: The detailed common comorbidity in premature infant in different Birth weight group.

## References

[1] Fan H.C., Ho L.I., Chi C.S., Cheng S.N., Juan C.J., Chiang K.L., Lin S.Z., Harn H.J. (2015). Current proceedings of cerebral palsy. Cell Transplant..

[2] Rosenbaum P. (2003). Cerebral palsy: What parents and doctors want to know. BMJ.

[3] Sadowska M., Sarecka-Hujar B., Kopyta I. (2020). Cerebral Palsy: Current Opinions on Definition, Epidemiology, Risk Factors, Classification and Treatment Options. Neuropsychiatr. Dis. Treat..

[4] Paneth N. (2023). Cerebral palsy as a public health indicator. Paediatr. Perinat. Epidemiol..

[5] Badawi N., McIntyre S., Hunt R.W. (2021). Perinatal care with a view to preventing cerebral palsy. Dev. Med. Child Neurol..

[6] Oskoui M., Coutinho F., Dykeman J., Jette N., Pringsheim T. (2013). An update on the prevalence of cerebral palsy: A systematic review and meta-analysis. Dev. Med. Child Neurol..

[7] Wu S.T., Lin C.H., Lin Y.H., Hsu Y.C., Hsu C.T., Lin M.C. (2024). Maternal risk factors for preterm birth in Taiwan, a nationwide population-based cohort study. Pediatr. Neonatol..

[8] Wang H.H., Hwang Y.S., Ho C.H., Lai M.C., Chen Y.C., Tsai W.H. (2021). Prevalence and Initial Diagnosis of Cerebral Palsy in Preterm and Term-Born Children in Taiwan: A Nationwide, Population-Based Cohort Study. Int. J. Environ. Res. Public Health.

[9] McIntyre S., Goldsmith S., Webb A., Ehlinger V., Hollung S.J., McConnell K., Arnaud C., Smithers-Sheedy H., Oskoui M., Khandaker G. (2022). Global prevalence of cerebral palsy: A systematic analysis. Dev. Med. Child Neurol..

[10] Chen R., Sjolander A., Johansson S., Lu D., Razaz N., Tedroff K., Villamor E., Cnattingius S. (2022). Impact of gestational age on risk of cerebral palsy: Unravelling the role of neonatal morbidity. Int. J. Epidemiol..

[11] Arnaud C., Ehlinger V., Delobel-Ayoub M., Klapouszczak D., Perra O., Hensey O., Neubauer D., Hollody K., Virella D., Rackauskaite G. (2021). Trends in Prevalence and Severity of Pre/Perinatal Cerebral Palsy Among Children Born Preterm From 2004 to 2010: A SCPE Collaboration Study. Front. Neurol..

[12] Chiang K.L., Huang C.Y., Fan H.C., Kuo F.C. (2019). Prolonged length of stay for acute hospital admissions as the increasing of age: A nationwide population study for Taiwan’s patients with cerebral palsy. Pediatr. Neonatol..

[13] Chiang K.L., Kuo F.C., Cheng C.Y., Chang K.P. (2019). Prevalence and demographic characteristics of comorbid epilepsy in children and adolescents with cerebral palsy: A nationwide population-based study. Childs Nerv. Syst..

[14] Hsieh C.Y., Su C.C., Shao S.C., Sung S.F., Lin S.J., Kao Yang Y.H., Lai E.C. (2019). Taiwan’s National Health Insurance Research Database: Past and future. Clin. Epidemiol..

[15] Chang M.J., Ma H.I., Lu T.H. (2015). Estimating the prevalence of cerebral palsy in Taiwan: A comparison of different case definitions. Res. Dev. Disabil..

[16] Murphy D.J., Hope P.L., Johnson A. (1997). Neonatal risk factors for cerebral palsy in very preterm babies: Case-control study. BMJ.

[17] Touyama M., Touyama J., Toyokawa S., Kobayashi Y. (2016). Trends in the prevalence of cerebral palsy in children born between 1988 and 2007 in Okinawa, Japan. Brain Dev..

[18] Kim S.W., Jeon H.R., Shin J.C., Youk T., Kim J. (2018). Incidence of Cerebral Palsy in Korea and the Effect of Socioeconomic Status: A Population-Based Nationwide Study. Yonsei Med. J..

[19] Chen C.C., Chen L.S., Yen M.F., Chen H.H., Liou H.H. (2012). Geographic variation in the age- and gender-specific prevalence and incidence of epilepsy: Analysis of Taiwanese National Health Insurance-based data. Epilepsia.

[20] Cremer N., Hurvitz E.A., Peterson M.D. (2017). Multimorbidity in Middle-Aged Adults with Cerebral Palsy. Am. J. Med..

[21] Heikkila K., Metsala J., Pulakka A., Nilsen S.M., Kivimaki M., Risnes K., Kajantie E. (2023). Preterm birth and the risk of multimorbidity in adolescence: A multiregister-based cohort study. Lancet Public Health.

[22] Chang Y.K., Tseng Y.T., Chen K.T. (2020). The epidemiologic characteristics and associated risk factors of preterm birth from 2004 to 2013 in Taiwan. BMC Pregnancy Childbirth.

[23] Laverty C., Surtees A., O’Sullivan R., Sutherland D., Jones C., Richards C. (2021). The prevalence and profile of autism in individuals born preterm: A systematic review and meta-analysis. J. Neurodev. Disord..

[24] Spittle A.J., Morgan C., Olsen J.E., Novak I., Cheong J.L.Y. (2018). Early Diagnosis and Treatment of Cerebral Palsy in Children with a History of Preterm Birth. Clin. Perinatol..

[25] Solaski M., Majnemer A., Oskoui M. (2014). Contribution of socio-economic status on the prevalence of cerebral palsy: A systematic search and review. Dev. Med. Child Neurol..

[26] Faramarzi R., Darabi A., Emadzadeh M., Maamouri G., Rezvani R. (2023). Predicting neurodevelopmental outcomes in preterm infants: A comprehensive evaluation of neonatal and maternal risk factors. Early Hum. Dev..

[27] Jadi J., Hyder S., Rodriguez Ormaza N.P., Twer E., Phillips M., Akinkuotu A., Reid T.D. (2023). Evaluation of Complications and Weight Outcomes in Pediatric Cerebral Palsy Patients With Gastrostomy Tubes. Am. Surg..

[28] Mehretie Y., Amare A.T., Getnet G.B., Mekonnen B.A. (2024). Length of hospital stay and factors associated with very-low-birth-weight preterm neonates surviving to discharge a cross-sectional study, 2022. BMC Pediatr..

[29] Vogel J.P., Chawanpaiboon S., Moller A.B., Watananirun K., Bonet M., Lumbiganon P. (2018). The global epidemiology of preterm birth. Best. Pract. Res. Clin. Obstet. Gynaecol..

[30] Keiser A.M., Salinas Y.D., DeWan A.T., Hawley N.L., Donohue P.K., Strobino D.M. (2019). Risks of preterm birth among non-Hispanic black and non-Hispanic white women: Effect modification by maternal age. Paediatr. Perinat. Epidemiol..

[31] Razaz N., Cnattingius S., Lisonkova S., Nematollahi S., Oskoui M., Joseph K.S., Kramer M. (2023). Pre-pregnancy and pregnancy disorders, pre-term birth and the risk of cerebral palsy: A population-based study. Int. J. Epidemiol..

[32] Sundelin H.E.K., Stephansson O., Johansson S., Ludvigsson J.F. (2020). Pregnancy outcome in women with cerebral palsy: A nationwide population-based cohort study. Acta Obstet. Gynecol. Scand..

