# Supplementary material for: The Prevalence and Risk Analysis of Cerebral Palsy and Other Neuro-Psychological Comorbidities in Children with Low Birth Weight in Taiwan: A Nationwide Population-Based Cohort Study
Source: J Clin Med. 2024 Jun 14;13(12):3480. doi: 10.3390/jcm13123480 (PMC11204667; doi:10.3390/jcm13123480)
Supplement: Supplementary file 1 [file jcm-13-03480-s001.zip › jcm-2974761-supplementary.pdf]

Supplementary Table 1 Sub-categories of enrollees of prematurity and their ICD 9 codes.

Sub-categories of preterm birth or low birth weight	ICD 9 codes
Extremely preterm (<1000g)	765.01, 765.02, 765.03, 765.11, 765.12, 765.13, V21.31, V21.32
Very preterm (1000-1499g)	765.04, 765.05, 765.14, 765.15, V21.33
Moderate preterm (500-1999g)	765.06, 765.07, 765.16, 765.17, V21.34
Late preterm (2000-2499g)	765.08, 765.18,V21.35

Supplementary Table2. Disease diagnostic coding of comorbidities.

**Disease diagnosis**	**ICD-9-CM**
Cerebral palsy	342 343 344
Pulmonary hypertension (Pulmonary circulation disease)	415 416 417
Acquired heart disease	420 421 422 423 424 425
Congenital heart disease	745 746 747
Congenital brain anomaly	742
Epilepsy	345 780.39
Hearing	389
Ophthalmic problems	360 361 362 363 364 365 366 367 368 369 370 371 372 373 374 375 376 377 378 379
Nutritional problem	783.0 783.1 783.2 783.3 783.7 783.9 307.1 307.50 3075.1 3075.2 3075.3 3075.4 3075.9
Pneumonia	480 481 482 483 484 485 486 487
Other respiratory infection excluded pneumonia	460 461 462 463 464 465 466 467 468 469 470 471 472 473 474 475 476 477 478
Respiratory failure	518
Respiratory distress syndrome in newborn	769
UTI	599.0 590 595
Gastrointestinal disorders	530 531 532 533 534 535 536 537 538 550 551 552 553 554 555 556 557 558 559 560 561 562 563 564 565 566 567 568 569 540 578 579 787 789
ADHD	314
Autism	299
Sleep disorders	307.4 347 780.5 327
Anxiety disorder	300
Acute stress reaction	308

Emotional disorders	313
Learning disability	31500 31501 31502 31509 3151 3152
Summation of developmental delay	3153 31531 31532 31539 3154 3155 3158 3159
-Speech or language disorder	3153 31531 31532 31539
-Developmental coordination disorder	3154
-Other specific delay in development	3158
-Mixed and unspecific delay	3155 3159
Intellectual disability	317 318 319
Hydrocephalus	3313 3314
Stroke	430 431 432 433 434 435 436 437 438
Dyskinesia	333
Tourette or tics	333.3 307.23
Personality disorder	301
Eating disorders	307.5
Behavioral disorders	312
Headache	784.0
Brain degeneration	330
Other Psychoses	290 291 292 293 294 295 296 297 298 300 307.20 307.21 307.22 307.23 307.3 313 316 333.3
CNS infection	320 321 322 323 324 325 326
Diseases of veins and lymphatics, and other diseases of circulator system	451 452 453 454 455 456 457 458 459
Febrile convulsion	780.31

Supplementary Table 3. The number and proportion of subsequent comorbidities in different birth weight groups of premature infants and the results of the chi-square test.

Comorbidity	Number (Proportion)					p
	<1000g	1000-1499g	1500-1999g	2000-2499g	Controls	
**Respiratory**	**1653**	**2765**	**2126**	**876**	**89288**	**< 0.001*** **
	**(73.76)**	**(61.98)**	**(40.57)**	**(27.05)**	**(58.82)**	
Respiratory distress syndrome in newborn	1362	2201	1329	389	467	< 0.001***
	0.6097	0.4935	0.2537	0.1202	0.0031	
Respiratory infections excluded pneumonia	476	795	820	428	83298	< 0.001***
	0.2131	0.1783	0.1565	0.1322	0.5487	
Pneumonia	202	198	137	82	37747	< 0.001***
	0.0904	0.0444	0.0262	0.0253	0.2486	
Respiratory failure	173	201	131	58	772	< 0.001***
	0.0774	0.0451	0.0250	0.0179	0.0051	
**Circulatory**	**906**	**1119**	**745**	**349**	**11553**	**< 0.001*** **
	**(40.43)**	**(25.08)**	**(14.22)**	**(10.77)**	**(7.61)**	
Congenital heart diseases	889	1073	694	304	10018	< 0.001***
	0.3979	0.2406	0.1325	0.0939	0.0660	
Heart failure or cardiomyopathy	21	67	80	55	2728	0.0106
	0.0094	0.015	0.0153	0.0170	0.0180	
Pulmonary hypertension (Pulmonary circulation diseases)	19	11	7	7	228	< 0.001***
	0.0085	0.0025	0.0013	0.0022	0.0015	
**Neurological**	**874**	**1075**	**986**	**172**	**20083**	**< 0.001*** **
	**(39.00)**	**(24.10)**	**(18.82)**	**(5.31)**	**(13.23)**	
Congenital brain	97	184	198	141	1065	< 0.001***

anomaly	0.0434	0.0413	0.0378	0.0436	0.0070	
Cerebral palsy	93	90	62	17	552	< 0.001***
	0.0416	0.0202	0.0118	0.0053	0.0036	
Hydrocephalus	24	17	9	4	301	< 0.001***
	0.0107	0.0038	0.0017	0.0012	0.0020	
~~Headache~~	~~0~~	~~0~~	~~0~~	~~0~~	~~461~~	-
	-	-	-	-	-	
Stroke	11	8	5	3	314	0.0064
	0.0049	0.0018	0.0010	0.0009	0.0021	
~~Brain degeneration~~	~~0~~	~~0~~	~~0~~	~~0~~	~~31~~	-
	-	-	-	-	-	
Ophthalmic problems	704	795	709	301	14720	< 0.001***
	0.3151	0.1783	0.1354	0.093	0.0970	
Hearing problem	43	64	39	10	2647	< 0.001***
	0.0192	0.0143	0.0074	0.0031	0.0174	
Epilepsy	39	42	40	22	2665	< 0.001***
	0.0192	0.0143	0.0074	0.0031	0.0174	
Movement disorders	3	7	4	1	1189	< 0.001***
	0.0013	0.0016	0.0008	0.0003	0.0078	
**Psycho-developmental**	**211**	**275**	**160**	**57**	**7369**	**< 0.001*** **
	**(9.42)**	**(6.16)**	**(3.05)**	**(1.76)**	**(4.85)**	
Developmental delay	203	256	152	54	5604	< 0.001***
	0.0909	0.0574	0.0290	0.0167	0.0369	
-Speech or language developmental disorder	32	41	32	14	3048	< 0.001***
	0.0143	0.0092	0.0061	0.0043	0.0201	
-Developmental	4	9	5	0	404	0.004

coordination disorder	0.0018	0.0020	0.0010	0.0000	0.0027	
-Other developmental disorder	25	47	28	13	715	< 0.001***
	0.0112	0.0105	0.0053	0.0040	0.0047	
-Mixed and unspecific delay	162	185	104	31	3226	< 0.001***
	0.0725	0.0415	0.0199	0.0096	0.0213	
Learning disability	2	3	0	0	90	0.2459
	0.0009	0.0007	0.0000	0.0000	0.0006	
~~Behavior problems~~	0	0	0	0	98	-
	-	-	-	-	-	
Childhood emotional disturbances	0	1	1	0	279	< 0.001***
	0.0000	0.0002	0.0002	0.0000	0.0018	
ADHD	5	8	7	3	985	< 0.001***
	0.0022	0.0018	0.0013	0.0009	0.0065	
Mental retardation	1	3	0	0	192	0.0122
	0.0004	0.0007	0.0000	0.0000	0.0013	
Autistic spectrum disorder	2	3	3	0	379	< 0.001***
	0.0009	0.0007	0.0006	0.0000	0.0025	
Anxiety (Neurosis)	0	1	1	0	138	0.0416
	0.0000	0.0002	0.0002	0.0000	0.0009	
~~Personality disorders~~	0	0	0	0	24	-
	-	-	-	-	-	
Acute stress reaction	1	0	0	0	38	0.4706
	0.0004	0.0000	0.0000	0.0000	0.0002	
**GI and Nutritional**	**571**	**806**	**791**	**411**	**61288**	**< 0.001***
	**(25.48)**	**(18.07)**	**(15.10)**	**(12.69)**	**(40.37)**	
Gastrointestinal problems	562	793	782	403	60684	< 0.001***
	0.2516	0.1778	0.1493	0.1245	0.3997	
Nutritional problems	18	26	15	9	2049	< 0.001***
	0.0081	0.0058	0.0029	0.0028	0.0135	
~~Eating disorders~~	0	0	0	0	26	-

	-	-	-	-	-	
**Urinary tract infection**	36	47	52	30	19283	< 0.001***
	0.0161	0.0105	0.0099	0.0093	0.1270	
**Sleep disorders**	2	2	1	0	308	< 0.001***
	0.0009	0.0004	0.0002	0.0000	0.0020	

* p < 0.05； ** p < 0.01； *** p < 0.001

Supplementary Table 4. The detailed common comorbidity in premature infant in different Birth weight group.

Comorbidities	Birth weight	n	OR	95% C.I.
**Respiratory**	**<1000g**	**1653**	**2.08**	(1.89, 2.28)
	**1000-1499g**	**2765**	**1.15**	(1.08, 1.22)
	**1500-1999g**	**2126**	**0.47**	(0.44, 0.50)
	**2000-2499g**	**876**	**0.24**	(0.22, 0.26)
	**Controls**	**89288**	**-**	**-**
Respiratory distress syndrome in newborn	<1000g	1362	621.30	(546.93, 705.79)
	1000-1499g	2201	358.25	(321.67, 399.00)
	1500-1999g	1329	112.68	(101.08, 125.63)
	2000-2499g	389	44.35	(38.65, 50.90)
	Controls	467	-	-
Respiratory infections excluded pneumonia	<1000g	476	0.22	(0.20, 0.24)
	1000-1499g	795	0.17	(0.16, 0.18)
	1500-1999g	820	0.15	(0.14, 0.16)
	2000-2499g	428	0.12	(0.10, 0.13)
	Controls	83298	-	-
Pneumonia	<1000g	202	0.30	(0.26, 0.34)
	1000-1499g	198	0.14	(0.12, 0.16)
	1500-1999g	137	0.08	(0.07, 0.09)
	2000-2499g	82	0.08	(0.06, 0.09)
	Controls	37747	-	-
Respiratory failure	<1000g	173	16.96	(14.29, 20.12)
	1000-1499g	201	9.34	(7.97, 10.93)
	1500-1999g	131	5.00	(4.14, 6.03)
	2000-2499g	58	3.50	(2.67, 4.58)
	Controls	772	-	-
**Circulatory**	**<1000g**	**906**	**8.56**	**(7.84, 9.34)**
	**1000-1499g**	**1119**	**4.15**	**(3.86, 4.45)**
	**1500-1999g**	**745**	**2.01**	**(1.85, 2.17)**
	**2000-2499g**	**349**	**1.46**	**(1.30, 1.63)**
	**Controls**	**11553**	**-**	**-**
Congenital heart diseases	<1000g	889	9.73	(8.92, 10.63)
	1000-1499g	1073	4.58	(4.27, 4.93)
	1500-1999g	694	2.15	(1.98, 2.34)
	2000-2499g	304	1.45	(1.30, 1.64)
	Controls	10018	-	-
Acquired heart diseases	<1000g	21	0.52	(0.34, 0.80)

	1000-1499g	67	0.84	(0.66, 1.06)
	1500-1999g	80	0.85	(0.68, 1.06)
	2000-2499g	55	0.94	(0.72, 1.23)
	Controls	2728	-	-
Pulmonary hypertension (Pulmonary circulation diseases)	<1000g	19	5.70	(3.58, 9.07)
	1000-1499g	11	1.65	(0.90, 3.02)
	1500-1999g	7	0.89	(0.42, 1.86)
	2000-2499g	7	1.44	(0.68, 3.06)
	Controls	228	-	-
**Neurological**	**<1000g**	**874**	**4.27**	**(3.92, 4.65)**
	**1000-1499g**	**1075**	**2.08**	**(1.94, 2.23)**
	**1500-1999g**	**986**	**1.53**	**(1.42, 1.64)**
	**2000-2499g**	**172**	**1.11**	**(1.01, 1.22)**
	**Controls**	**20083**	**-**	**-**
Congenital brain anomaly	<1000g	97	6.55	(5.30, 8.09)
	1000-1499g	184	6.14	(5.24, 7.20)
	1500-1999g	198	5.54	(4.75, 6.47)
	2000-2499g	141	6.35	(5.31, 7.59)
	Controls	1065	-	-
Cerebral palsy	<1000g	93	11.80	(9.42, 14.79)
	1000-1499g	90	5.53	(4.41, 6.93)
	1500-1999g	62	3.36	(2.57, 4.38)
	2000-2499g	17	1.47	(0.90, 2.39)
	Controls	552	-	-
Hydrocephalus	<1000g	24	5.63	(3.71, 8.54)
	1000-1499g	17	1.93	(1.18, 3.16)
	1500-1999g	9	0.87	(0.44, 1.70)
	2000-2499g	4	0.61	(0.22, 1.68)
	Controls	301	-	-
Stroke	<1000g	11	2.44	(1.34, 4.44)
	1000-1499g	8	0.87	(0.43, 1.74)
	1500-1999g	5	0.46	(0.19, 1.11)
	2000-2499g	3	0.44	(0.14, 1.33)
	Controls	314	-	-
Ophthalmic problems	<1000g	704	4.34	(3.96, 4.75)
	1000-1499g	795	2.02	(1.87, 2.19)
	1500-1999g	709	1.46	(1.35, 1.58)
	2000-2499g	301	0.94	(0.84, 1.06)
	Controls	14720	-	-

Hearing problems	<1000g	43	1.12	(0.83, 1.51)
	1000-1499g	64	0.82	(0.64, 1.05)
	1500-1999g	39	0.42	(0.31, 0.58)
	2000-2499g	10	0.17	(0.10, 0.31)
	Controls	2647	-	-
Epilepsy	<1000g	39	1.01	(0.74, 1.39)
	1000-1499g	42	0.53	(0.39, 0.72)
	1500-1999g	40	0.43	(0.32, 0.59)
	2000-2499g	22	0.38	(0.25, 0.57)
	Controls	2665	-	-
Movement disorders	<1000g	3	0.17	(0.06, 0.54)
	1000-1499g	7	0.20	(0.10, 0.41)
	1500-1999g	4	0.10	(0.04, 0.25)
	2000-2499g	1	0.04	(0.01, 0.17)
	Controls	1189	-	-
**Psycho-developmental**	**<1000g**	**211**	**2.09**	**(1.82, 2.41)**
	**1000-1499g**	**275**	**1.27**	**(1.12, 1.44)**
	**1500-1999g**	**160**	**0.62**	**(0.53, 0.73)**
	**2000-2499g**	**57**	**0.35**	**(0.27, 0.45)**
	**Controls**	**7369**	**-**	**-**
Developmental delay	<1000g	203	2.69	(2.32, 3.12)
	1000-1499g	256	1.58	(1.39, 1.80)
	1500-1999g	152	0.78	(0.67, 0.92)
	2000-2499g	54	0.43	(0.33, 0.56)
	Controls	5604	-	-
-Speech or language developmental disorder	<1000g	32	0.73	(0.51, 1.04)
	1000-1499g	41	0.45	(0.33, 0.60)
	1500-1999g	32	0.30	(0.21, 0.43)
	2000-2499g	14	0.21	(0.12, 0.35)
	Controls	3048	-	-
-Developmental coordination disorder	<1000g	4	0.69	(0.27, 1.74)
	1000-1499g	9	0.75	(0.40, 1.42)
	1500-1999g	5	0.36	(0.15, 0.85)
	2000-2499g	0	0.00	(0.00, 0.00)
	Controls	404	-	-
-Other developmental disorder	<1000g	25	2.44	(1.64, 3.63)
	1000-1499g	47	2.25	(1.67, 3.02)
	1500-1999g	28	1.14	(0.79, 1.66)

	2000-2499g	13	0.83	(0.49, 1.41)
	Controls	715	-	-
-Mixed and unspecific delay	<1000g	162	3.70	(3.14, 4.36)
	1000-1499g	185	1.99	(1.71, 2.31)
	1500-1999g	104	0.94	(0.77, 1.14)
	2000-2499g	31	0.44	(0.31, 0.62)
	Controls	3226	-	-
Learning disability	<1000g	2	1.49	(0.37, 6.00)
	1000-1499g	3	1.11	(0.36, 3.45)
	1500-1999g	0	0.00	(0.00, 0.00)
	2000-2499g	0	0.00	(0.00, 0.00)
	Controls	90	-	-
Childhood emotional disturbances	<1000g	0	0.00	(0.00, 0.00)
	1000-1499g	1	0.12	(0.02, 0.68)
	1500-1999g	1	0.11	(0.02, 0.57)
	2000-2499g	0	0.00	(0.00, 0.00)
	Controls	279	-	-
ADHD	<1000g	5	0.33	(0.13, 0.79)
	1000-1499g	8	0.25	(0.13, 0.50)
	1500-1999g	7	0.21	(0.10, 0.44)
	2000-2499g	3	0.15	(0.05, 0.44)
	Controls	985	-	-
Mental retardation	<1000g	1	0.34	(0.05, 2.26)
	1000-1499g	3	0.51	(0.17, 1.54)
	1500-1999g	0	0.00	(0.00, 0.00)
	2000-2499g	0	0.00	(0.00, 0.00)
	Controls	192	-	-
Autistic spectrum disorder	<1000g	2	0.37	(0.10, 1.34)
	1000-1499g	3	0.26	(0.09, 0.78)
	1500-1999g	3	0.23	(0.08, 0.70)
	2000-2499g	0	0.00	(0.00, 0.00)
	Controls	379	-	-
Anxiety (Neurosis)	<1000g	0	0.00	(0.00, 0.00)
	1000-1499g	1	0.23	(0.03, 1.67)
	1500-1999g	1	0.22	(0.03, 1.58)
	2000-2499g	0	0.00	(0.00, 0.00)
	Controls	138	-	-
Acute stress reaction	<1000g	1	1.80	(0.25, 13.24)
	1000-1499g	0	0.00	(0.00, 0.00)

	1500-1999g	0	0.00	(0.00, 0.00)
	2000-2499g	0	0.00	(0.00, 0.00)
	Controls	38	-	-
**GI and nutritional**	**<1000g**	**571**	**0.51**	**(0.47, 0.57)**
	**1000-1499g**	**806**	**0.32**	**(0.30, 0.35)**
	**1500-1999g**	**791**	**0.26**	**(0.24, 0.28)**
	**2000-2499g**	**411**	**0.21**	**(0.19, 0.23)**
	**Controls**	**61288**	**-**	**-**
Gastrointestinal problems	<1000g	562	0.51	(0.47, 0.56)
	1000-1499g	793	0.32	(0.30, 0.35)
	1500-1999g	782	0.26	(0.24, 0.28)
	2000-2499g	403	0.20	(0.18, 0.23)
	Controls	60684	-	-
Nutritional problems	<1000g	18	0.60	(0.38, 0.95)
	1000-1499g	26	0.43	(0.29, 0.63)
	1500-1999g	15	0.21	(0.13, 0.34)
	2000-2499g	9	0.20	(0.11, 0.37)
	Controls	2049	-	-
Urinary tract infection	<1000g	36	0.11	(0.08, 0.16)
	1000-1499g	47	0.07	(0.06, 0.10)
	1500-1999g	52	0.07	(0.05, 0.09)
	2000-2499g	30	0.06	(0.04, 0.09)
	Controls	19283	-	
Sleep problems	<1000g	2	0.44	(0.11, 1.67)
	1000-1499g	2	0.22	(0.06, 0.82)
	1500-1999g	1	0.10	(0.02, 0.57)
	2000-2499g	0	0.00	(0.00, 0.60)
	Controls	308	-	-
